# Expansion of the *TLO* gene family enhances the virulence of *Candida* species

**DOI:** 10.1371/journal.pone.0200852

**Published:** 2018-07-20

**Authors:** Peter R. Flanagan, Jessica Fletcher, Hannah Boyle, Razvan Sulea, Gary P. Moran, Derek J. Sullivan

**Affiliations:** 1 Microbiology Research Unit, Division of Oral Biosciences, Dublin Dental University Hospital, Dublin, Ireland; 2 University of Dublin, Trinity College Dublin, Dublin, Ireland; Newcastle University, UNITED KINGDOM

## Abstract

The *TLO* genes are a family of subtelomeric ORFs in the fungal pathogens *Candida albicans* and *C*. *dubliniensis* encoding a subunit of the Mediator complex homologous to Med2. The more virulent pathogen *C*. *albicans* has 15 copies of the gene whereas the less pathogenic species *C*. *dubliniensis* has only two. To investigate if expansion of the *TLO* repertoire in *C*. *dubliniensis* has an effect on phenotype and virulence we expressed three representative *C*. *albicans TLO* genes (TLOβ2, *TLOγ11* and TLOα12) in a wild type *C*. *dubliniensis* background, under the control of either their native or the *ACT1* promoter. Expression of *TLO*β*2* resulted in a hyperfilamentous phenotype, while overexpression of *TLOγ11* and *TLO*α*12* resulted in enhanced resistance to oxidative stress. Expression of all three *TLO* genes from the *ACT1* promoter resulted in increased virulence in the *Galleria* infection model. In order to further investigate if individual *TLO* genes exhibit differences in function we expressed six representative *C*. *albicans TLO* genes in a *C*. *dubliniensis Δtlo1/Δtlo2* double mutant. Differences were observed in the ability of the expressed *CaTLO*s to complement the various phenotypes of the mutant. All *TLO* genes with the exception of *TLOγ7* could restore filamentation, however only *TLO*α*9*, γ*11* and α*12* could restore chlamydospore formation. Differences in the ability of *CaTLO* genes to restore growth in the presence of H_2_O_2_, calcofluor white, Congo red and at 42°C were observed. Only *TLO*α*3* restored wild-type levels of virulence in the *Galleria* infection model. These data show that expansion of the *TLO* gene family in *C*. *dubliniensis* results in gain of function and that there is functional diversity amongst members of the gene family. We propose that this expansion of the *TLO* family contributes to the success of *C*. *albicans* as a commensal and opportunistic pathogen.

## Introduction

*Candida* species (spp.) are an important component of the human microbiota. They are found in a wide range of anatomic niches, particularly in the gastrointestinal and vaginal tracts and if host conditions provide an opportunity they can evade immune responses and cause a spectrum of diseases, ranging from superficial infections of the mucosae to life threatening systemic infections in severely immunocompromised patients. In particular, *Candida* spp. have been cited as the fourth most common cause of nosocomial bloodstream infections [1].

The most pathogenic *Candida* species are *C*. *albicans*, *C*. *parapsilosis*, *C*. *tropicalis* and *C*. *glabrata*. [2]. *C*. *albicans* is by far the most commonly identified cause of candidiasis and is often regarded as the most pathogenic fungal species in humans. *C*. *albicans* is a highly versatile microorganism that has the ability to activate rapid transcriptional responses in order to adapt to changing environmental conditions, potentially allowing it to colonise and infect multiple anatomic sites [3].

One of the reasons why *C*. *albicans* is more pathogenic than other *Candida* spp. is that, apart from *Candida dubliniensis*, it is the only truly dimorphic *Candida* species, having the ability to switch between yeast and filamentous forms of growth [4]. Hyphal cells and hypha-specific proteins are well documented contributors to virulence and facilitate adherence to the host, penetration of tissues and the formation of biofilms [5–7].

**Fig 1:**
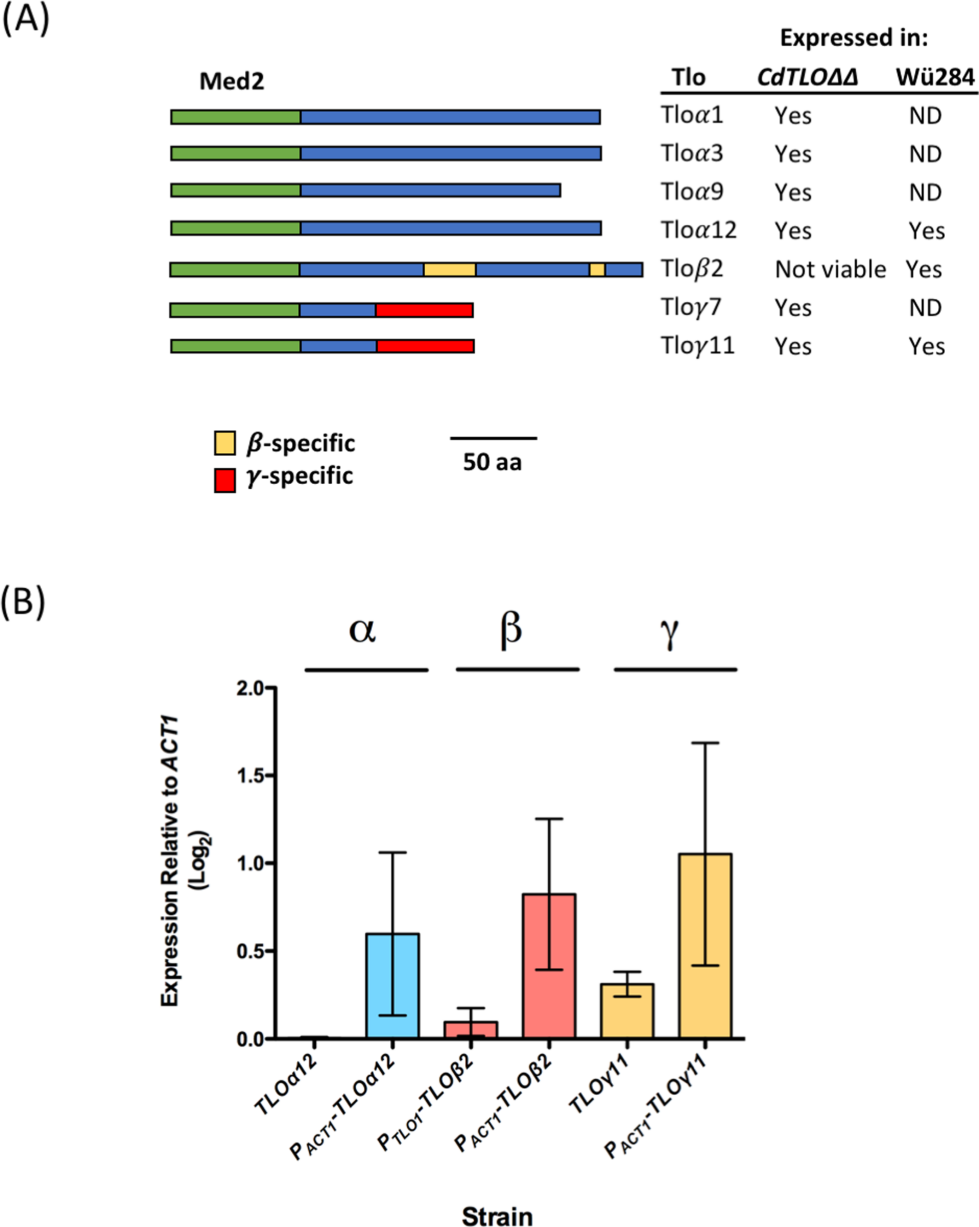
Structure and expression of *C*. *albicans TLO* genes. (A) Diagram comparing the structure of the Tlo proteins analysed in this study, based on the models of Anderson *et al*. [11]. The green box represents the conserved Med2-like domain. The blue box represents the clade-specific c-terminus. The γ- and β-specific regions are indicated by yellow and red boxes, respectively. The table on the right indicates which genes have been expressed in wild-type *C*. *dubliniensis* Wü284 and the *TLO* null derivative *ΔΔtlo*. ND = not done. (B) RT-PCR expression data of *C*. *albicans TLO*s expressed in the *C*. *dubliniensis* WT Wü284 strain. RT-PCR expression graphs represent the results of three independent experiments.

C*andida dubliniensis*, which was first identified in 1995 [8] is very closely related to *C*. *albicans*. The genomes of both species are highly similar (i.e. 98% synteny [9]) and they share many phenotypic traits, including the capacity to form hyphae. Despite their very close relationship *C*. *dubliniensis* has been shown to be far less virulent than *C*. *albicans* and is only rarely found to be the cause of systemic infections [10]. Comparison of the genomes to identify the underlying genetic differences for the disparity in virulence found gene family size as the primary differentiating feature. Some of these differences were found in gene families known to contribute to virulence (e.g. the agglutinin-like sequence (*ALS*) and secretory aspartyl proteinase (*SAP*) gene families), however, one of the greatest differences was observed in the composition of the *TLO* (TeLOmere-associated) family in each species. The *C*. *albicans* SC5314 genome contains 15 *TLO* genes compared with just two in *C*. *dubliniensis* [9]. In *C*. *albicans*, as the name suggests, the *TLO* genes are situated close to the telomeres of each chromosome. The *C*. *albicans TLO* genes can be divided into four distinct clades based on the structure of their genes (Fig 1A). These include the relatively highly expressed α clade containing six members, a single β clade gene, the γ clade containing seven members, and the ψ clade containing a single pseudogene member [11–13]. *TLO* copy number varies between strains (10–15), with variation in the number of α and γ clade genes [14]. However, a single β clade gene is present in all genomes analysed to date [14]. The N-terminus of the *C*. *albicans TLO* genes encodes a conserved Med2 domain and these genes are now known to encode the Med2 component of the Mediator complex [11,15]. Mediator is a large multi-subunit protein complex which is conserved throughout eukaryotes and mediates interaction between RNA polymerase II and the machinery used in the initiation of transcription at target gene promoters [16,17]. Uwamahoro *et al*. (2012) demonstrated that *C*. *albicans* Mediator has a role in the expression of genes related to virulence traits [17] and it has also recently been demonstrated to play a role in resistance to antifungal drugs [18,19]. Tlo/Med2 forms a part of the Tail module of Mediator, along with Med3 and Med15 [15]. Different Tlos are found at different levels in *C*. *albicans* and *C*. *dubliniensis* [15] suggesting that there are pools of Mediator in each species with a different Med2 component. Given the size of the *TLO* family in *C*. *albicans* it has also been proposed that there is a substantial pool excess of “Mediator-free” Tlo in this species [11].

Deletion of the two *TLO* genes in *C*. *dubliniensis* resulted in defects in activation of transcriptional responses associated with a number of virulence traits including tolerance of oxidative stress and hypha formation [12], while overexpression of *CdTLO2* (and creation of a pool of “free” Tlo), but not *CdTLO1*, in *C*. *dubliniensis* results in hyperfilamentation [20]. As well as confirming the role of Med2 in virulence, the data from *C*. *dubliniensis* suggest differences in functionality amongst the two Tlo proteins expressed in that species.

We have previously proposed that the increased virulence of *C*. *albicans* compared to other *Candida* species may be due to an increased transcriptional flexibility due to its expanded family of Tlo proteins which may have differences in functionality [12]. Evidence that individual Tlo proteins have specific function(s) in *C*. *albicans* has recently been provided by Dunn *et al*. [21] who investigated the phenotypic effect of controlling the expression of individual *TLO* genes using a Tet-ON system. In order to investigate our hypothesis, we have added to the repertoire of *TLO* genes in *C*. *dubliniensis* by heterologously expressing representative *C*. *albicans TLO* genes in a wild-type *C*. *dubliniensis* strain. The rationale for selecting *C*. *dubliniensis* as the host species was due to the low copy number of native *TLO* genes in this species as we reasoned that it could be difficult to determine a phenotypic effect in a *C*. *albicans* background of 15 *TLO* genes. In addition, we also attempted to investigate the functional diversity within the *C*. *albicans TLO* gene family, by expressing *TLO* representative *CaTLO* genes in a *C*. *dubliniensis ΔΔtlo* double mutant and identified the phenotypes conferred by each gene. We propose that our data demonstrate possible evolutionary advantages associated with *TLO* gene duplication and diversification.

## Materials & methods

### *Candida* strains & culture conditions

The strains of *Candida* spp. used in this study and their genotypes are listed in S1 Table. All *Candida* strains were routinely grown on Yeast Extract Peptone Dextrose (YEPD) agar at 37°C. Nourseothricin-resistant transformants were cultured on YEPD agar containing nourseothricin [100 μg/ml (NAT100)]. Lee’s Medium [22] and Spider medium [23] were used to induce filamentation. Cornmeal agar supplemented with 1% Tween® 80 was used for chlamydospore formation.

For spot plate assay experiments, a suspension of 2 x 10^6^ cells/ml was prepared from overnight cultures and 7 μl from serial dilutions (10^0^ to 10^−4^) were spotted onto YEPD agar plates containing the indicated agents. The plates were incubated at 37°C for 48 h in a static incubator. Growth was recorded using a Flash n’ Go plate visualizer (IUL Instruments). Each experiment was carried out on three separate occasions.

For liquid culture, YEPD broth was used in an orbital incubator at 200 r.p.m. at the indicated temperature. In order to determine the doubling times of strains, the optical densities of cultures were measured at 600nm during the exponential growth phase and plotted using Prism GraphPad (GraphPad, CA, USA). Doubling times were calculated from 3 replicate growth curves. Galactose (2% w/v) was substituted for glucose where indicated. Induction of filamentation in liquid cultures was carried out with cells from overnight YEPD broth cultures grown at 30°C, which were washed twice with sterile Milli-Q water (Millipore Ireland B.V., Co. Cork, Ireland) and added to hyphal-induction medium (10% v/v foetal bovine serum in dH_2_O) to a density of 2 x 10^5^ cells/ml in a six-well tissue culture plate at 37°C. The numbers of true hyphal cells were quantified using a Nikon E600 microscope and a Nikon TMS-F inverted light microscope (Nikon U.K., Surrey, U.K.). Experiments were carried out on three separate occasions.

### Heterologous expression of *C*. *albicans TLO* genes in *C*. *dubliniensis*

*C*. *albicans TLO* genes β*2*, γ*11* and α*12* were heterologously expressed in the *C*. *dubliniensis* wild type strain Wü284. *TLO*γ*11* and α*12* were expressed under the control of their native promoters, however the *TLO*β*2* promoter sequence is incomplete in the SC5314 genome sequence so a fusion gene with the *TLO*α*1* promoter (S1 File) was synthesised by GeneWiz (Essex, UK) and inserted in the *Xho*I and *Hin*dIII restriction endonuclease sites of pCDRI [24]. *TLO*γ*11* and α*12* were amplified from SC5314 using gene-specific primers (S2 Table) containing recognition sequences for *Xho*I and *Hin*dIII restriction endonucleases. Digested amplimers were ligated to *Xho*I/*Hin*dIII cut pCDRI plasmid using T4 DNA ligase (Promega, Wisconsin, USA) and transformed into *E*. *coli* XL10 competent cells (Sigma-Aldrich, Missouri, USA) using standard protocols. Transformants were selected on pre-warmed Lysogeny (L) agar supplemented with 100 μg/ml ampicillin. Plasmid pCDRI and its derivatives were linearised with *Eco*47III and transformed in *C*. *dubliniensis* as described by Staib *et al*. [25]. Transformants were selected on YEPD agar containing 100 μg/ml nourseothricin. Additional plasmid constructs containing *TLO*s β*2*, γ*11* and α*12* were also generated in pGM161, which is a derivative of pCDRI allowing expression from the *ACT1* promoter, using the same cloning strategy [24].

*C*. *albicans TLO* genes α*1*, α*3*, γ*7*, α*9*, γ*11* and α*12* were heterologously expressed in the *C*. *dubliniensis* ΔΔ*tlo* double mutant under the control of their native promoters. Each gene was PCR amplified and cloned in pCDRI and introduced in the ΔΔ*tlo* double mutant as described above.

### cDNA synthesis and qualitative real-time PCR

RNA was extracted and used to generate cDNA as described by Flanagan *et al*. [26]. qRT-PCR was carried out on the Applied Biosystem 7500 Fast Real Time PCR System as described by Flanagan *et al*. [26]. Plates were set up in triplicate with the endogenous control, *ACT1*, run alongside each target. Results were exported into Microsoft Excel and the delta Ct values calculated for each sample. These were ultimately graphed using GraphPad Prism version 6 (San Diego, California, USA, www.graphpad.com).

### Biofilm induction assays

Biofilm mass was determined using crystal violet to quantify biomass. Cells were grown in YEPD at 37°C overnight with shaking at 200 rpm. Following overnight incubation, 100 ml was removed and transferred to YNB with 100 mM glucose and incubated overnight at 37°C with shaking at 200 rpm. Following the second night of incubation, cells were washed in 1X PBS and resuspended in 1 ml of YNB with 100 mM glucose at a cell density of 2 x 10^6^ cells/ml. 100 μl of each strain was placed in triplicate into a 96 well plate and incubated at 37°C for 90 min. Following incubation, the medium was aspirated, and cells washed twice with 150 ml 1X PBS. A 100 μl volume of YNB containing 100 mM glucose was placed onto the washed cells and the plates were incubated at 37°C for 24 and 48 hr. Following incubation, the wells were washed three times with 200 μl sterile 1X PBS to remove non-adherent cells and 110 μl of 0.4% (v/v) crystal violet was added to each well and stained at room temperature for 45 min. The crystal violet was removed and each well washed with 200 μl of dH_2_O three times. The wells were de-stained with 200 μl of 95% (v/v) ethanol for 45 min. A 100 μl aliquot of each suspension was transferred to a new 96-well plate and the absorbance measured at an OD_540_ using a Tecan Plate Reader system (Tecan). Results were analysed using GraphPad Prism version 6.

### *In vivo* infection model

Candidal virulence was assessed using the wax moth larva *Galleria mellonella* obtained from Live Foods Direct (Sheffield, England). Larvae were stored at 15°C in wood shavings in the dark prior to use and those that weighed between 0.20 to 0.30 g were used within 2 weeks of receipt. For each infection experiment, 10 larvae were placed into sterile 9 cm petri dishes lined with Whatman filter paper and wood shavings. *Candida* strains were assigned a random code prior to each experiment to facilitate blind assessment of virulence. For infection, 1 X 10^6^ yeast cells in 20 μl were injected into the haemocoel via the last left pro-leg with a 30G insulin U-100 Micro-Fine syringe (BD New Jersey, USA) as described by Cotter *et al*. (2000) [27]. The inoculated larvae were incubated at 30°C and larval mortality was assessed at 24 h intervals, as described by Cotter *et al*. (2000) [27]. The results were analysed using GraphPad Prism version 6.

## Results

### Expression of *Candida albicans TLO* genes in wild type *C*. *dubliniensis*

In order to investigate the effect on phenotype of expanding the repertoire of *TLO* genes in *C*. *dubliniensis*, we expressed the *C*. *albicans TLO*β*2*, *TLO*γ*11* and *TLO*α*12* genes (representing each of the three *CaTLO* clades) in the *C*. *dubliniensis* WT Wü284 background under the control of a native *TLO* promoter and that of the constitutively expressed *ACT1* gene. In the case of *TLO*β*2* for which no promoter sequence was available, we used the *TLO*α*1* promoter as a proxy native promoter as both *TLO*β*2* and *TLO*α*1* exhibit similar mRNA expression levels [11].

Quantitative Real Time PCR was used to determine the level of expression of each *TLO* under the expression of their native promotor and that of the *ACT1* gene (Fig 1B). The level of each *TLO* expressed under their native promotor was lower compared with that of the *ACT1* gene. *TLO*β*2*, under the expression of the native *TLO*α*1* promoter, was expressed at 0.1 relative to *ACT1*. When placed under the expression of the *ACT1* gene, the expression increased to 1.31 relative to *ACT1*, a fold-change of 13.1. *TLO*γ*11* under the control of its native and the *ACT1* promoters was expressed at 0.45 and 1.05, respectively, relative to *ACT1*, a fold-change of 2.3. Similarly, *TLO*α*12* under the control of the native promoter and *ACT1* gene showed expression levels of 0.005 and 0.5 relative to *ACT1*, a fold-change of 118.

Once the level of expression of each gene had been determined, a range of phenotypic tests was then performed to determine whether the expression of additional *C*. *albicans TLO* genes had the ability to affect the phenotype of the host strain.

### *TLO*β*2* expression in wild type *C*. *dubliniensis* results in hyperfilamentous growth

**Fig 2:**
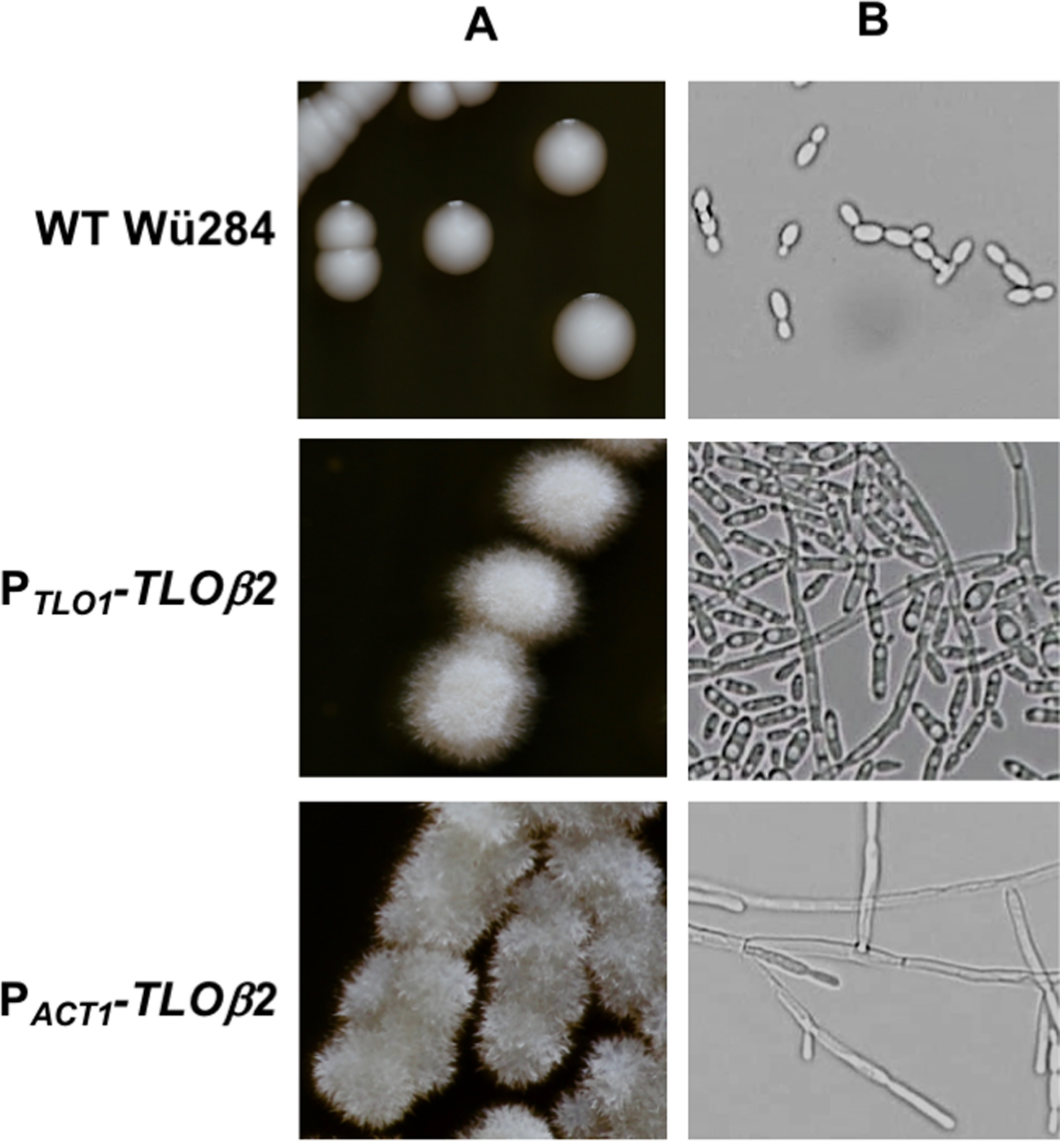
*C*. *albicans TLO*β*2* confers filamentous growth in *C*. *dubliniensis*. Colony (A) and cellular (B) morphology of *C*. *dubliniensis* WT Wü284 and derivatives harboring *TLO*β*2* expressed from the *TLO1* promoter (P_*TLO1*_*-TLO*β*2*) and the *ACT1* promoter (P_*ACT1*_*-TLO*β*2*). Colonies were grown for 48 h on solid YEPD agar. The morphology of the cells in representative colonies of each derivative was visualised using a x40 objective lens.

In wild type *C*. *dubliniensis* Wü284, expression of *TLO*γ*11* and *TLO*α*12* under the native or *ACT1* promoter did not affect the colony morphology of the strain on YEPD agar. However, *TLO*β*2* whether expressed under the comparatively weak *TLO*α*1* promoter or the *ACT1* promoter in wild-type *C*. *dubliniensis* resulted in wrinkled colonies on YEPD agar and hypha formation in YEPD broth (Fig 2). This phenotype was affected by expression levels of *TLO*β*2*, with the *TLO*α*1* promoter variant exhibiting a predominantly pseudohyphal mode of growth in YEPD broth, and the *ACT1* variant producing longer filaments with evidence of true hyphal growth (Fig 2B).

### *TLO* expansion in wild type *C*. *dubliniensis* affects growth rate

**Fig 3:**
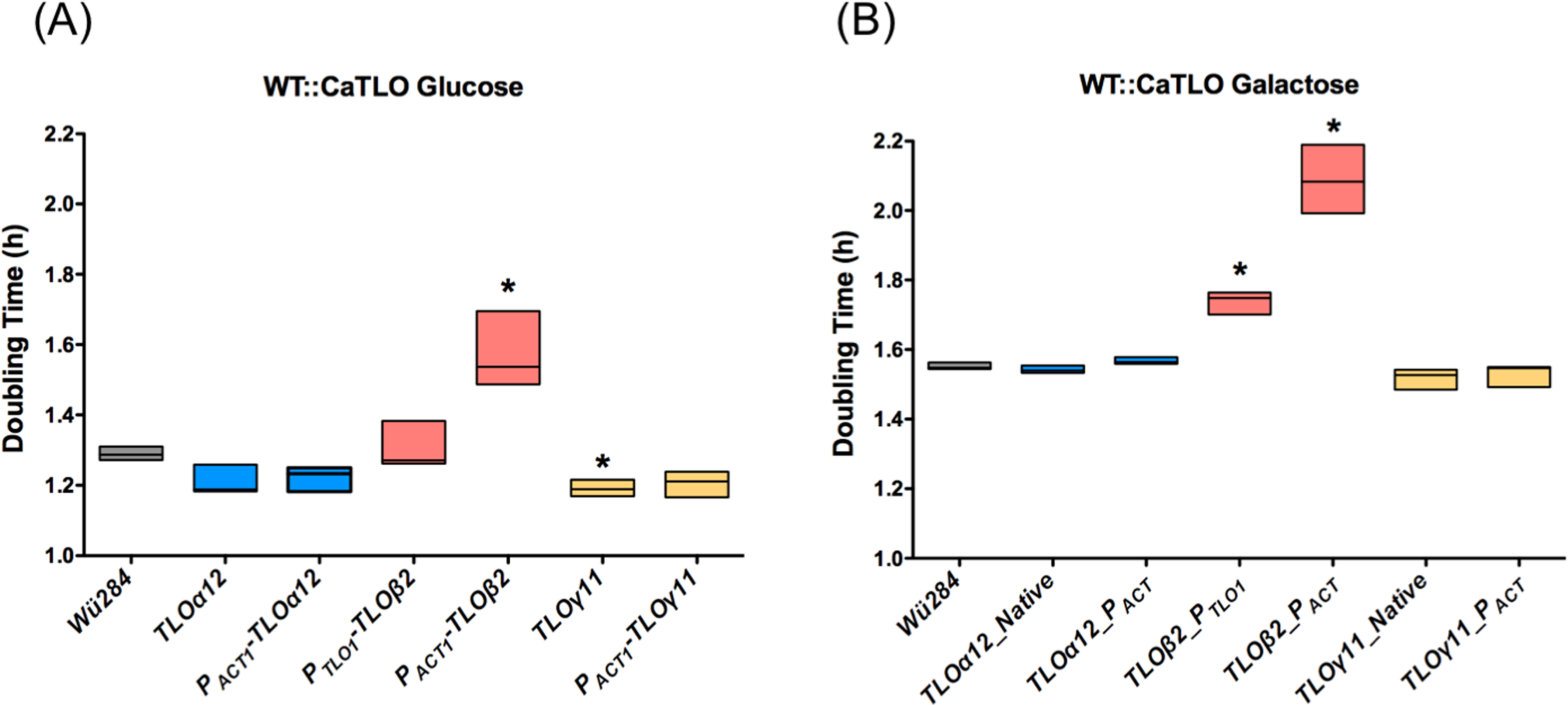
The effect of *C*. *albicans TLO* genes on growth rates in YEP-Glucose and -Galactose broth. Doubling times of WT Wü284 and derivatives expressing the indicated *C*. *albicans TLO* genes in YEP-Glucose (A) and -Galactose (B). Asterisks indicate significant difference from Wü284. Data were generated in three replicate experiments.

It has previously been shown that deletion of the two *TLO* genes present in the *C*. *dubliniensis* genome leads to reduced growth rate in YEPD and also results in greatly increased doubling times when galactose is the sole source of carbon [12]. To investigate the ability of an expanded *TLO* gene repertoire to affect growth rate we cultured all strains in YEP-Glucose and YEP-Galactose over an 8 h time course. In WT *C*. *dubliniensis* Wü284, expression of *TLOα12* under the control of its native or the *ACT1* promoter did not affect doubling times in YEP-Glucose or YEP-Galactose (Fig 3). Expression of *TLOγ11* under its native promoter in WT Wü284 reduced the doubling time in YEP-Glucose by approximately 10 min (Fig 3A). Expression of *TLOβ2* had the effect of greatly reducing growth in both media, and this was most significant in strain P_*ACT1*_*-TLOβ2* compared to P_*TLO1*_*-TLOβ2* (Fig 3B). This effect on growth rate is most likely due to the filamentous morphology exhibited by these strains (Fig 2).

### *TLO* expansion in wild type *C*. *dubliniensis* enhances H_2_O_2_ resistance

**Fig 4:**
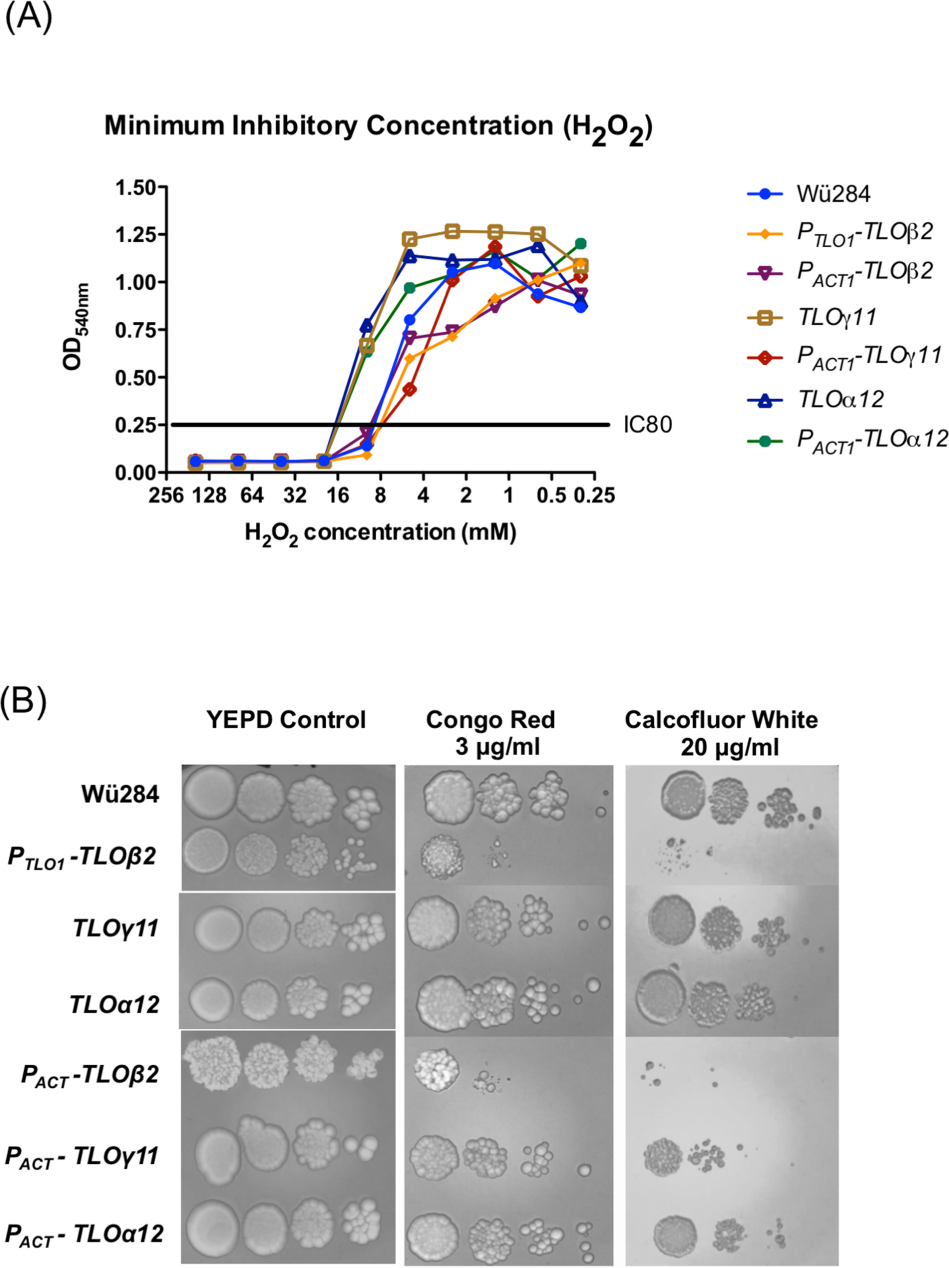
The effect of *C*. *albicans TLO* genes on susceptibility to H_2_O_2_ and cell wall damaging agents. (A) Minimum inhibitory concentration of H_2_O_2_ was determined by broth dilution. The IC_80_ is indicated and shows the concentration of H_2_O_2_ that reduced growth of the derivatives tested below 80% of the inhibitor-free control. (B) Ten-fold serial dilutions (left to right) of 2 x 10^4^ cells were spotted on to plates containing 3 μg/ml Congo Red and 20 μg/ml Calcofluor White.

Using a broth dilution MIC test for H_2_O_2_ we showed that expression of TLOγ11 and *TL*Oα*12* in WT *C*. *dubliniensis* Wü284 under their native promoters led to a doubling of MIC_80_ from 10mM to 20mM. *TL*O*α12* expressed using the *ACT1* promoter also led to a similar increase in MIC_80_. Using this assay, expression of *TLO*β*2* under the control of the *TLO*α*1* or *ACT1* promoters did not affect susceptibility to H_2_O_2_ (Fig 4A).

### *TLO*β*2* increases susceptibility to cell wall damaging agents

In order to determine if the *C*. *albicans TLO* genes differ in their effects on cell wall stress responses we compared the effect of these genes on growth on media containing the β-1,3-glucan-binding dye Congo Red and the chitin-binding dye Calcofluor White. Expression of *TL*Oγ*11 and TL*O*α12* in WT *C*. *dubliniensis* Wü284 under their native promoters did not affect susceptibility to Congo Red or Calcofluor White, while expression of *TLO*β*2* under the control of the *TLO*α*1* or *ACT1* promoters in wild type *C*. *dubliniensis* resulted in increased susceptibility to both agents (Fig 4B).

### Biofilm formation

**Fig 5:**
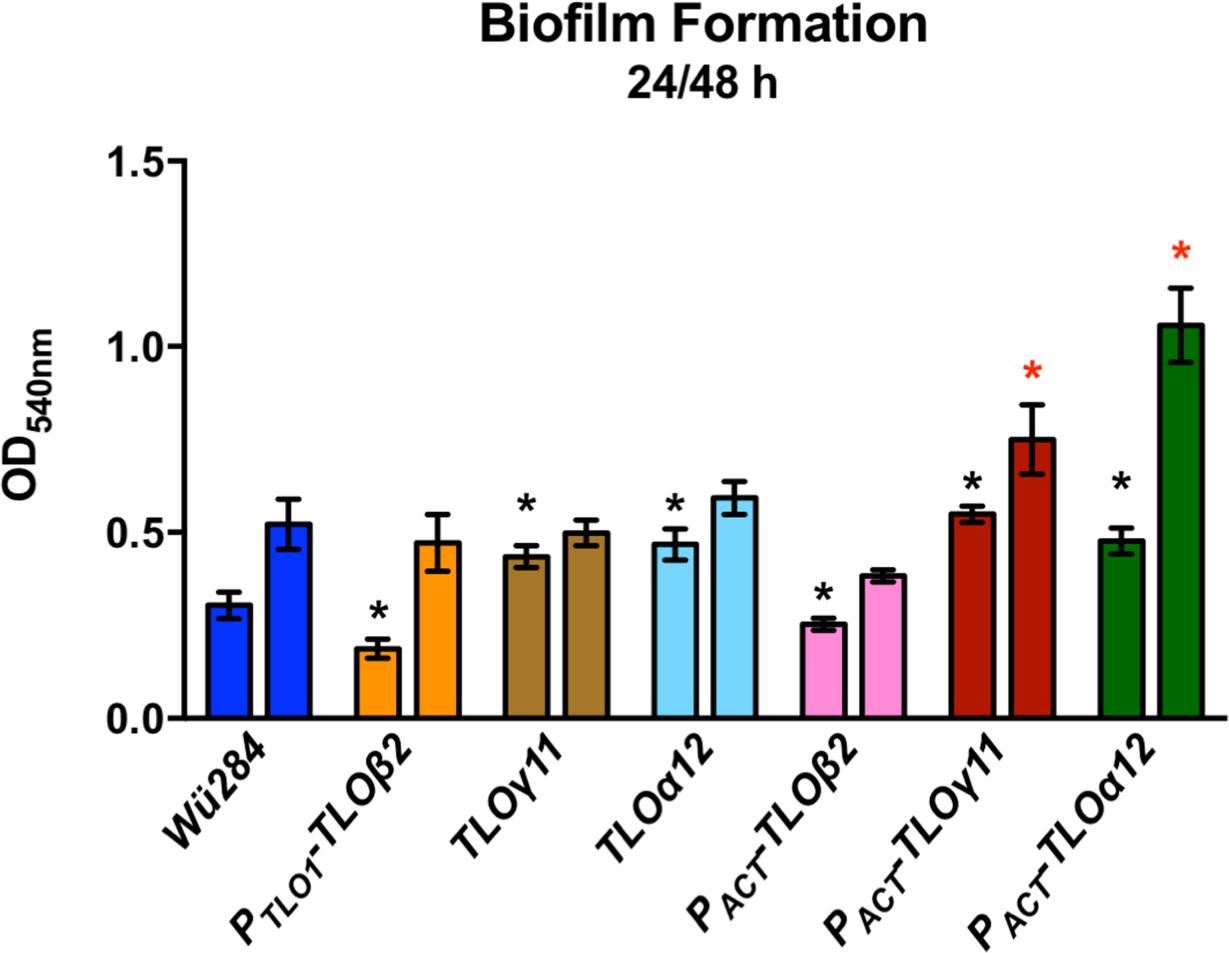
The effect of *C*. *albicans TLO* genes on biofilm formation on plastic surfaces. Each strain was grown in Spider medium in a 96-well plate for 48 h and biomass measured using a Crystal Violet assay. An asterisk indicates significant differences with * at 24 h and red * at 48 h.

The ability to form biofilm on plastic surfaces following 24 h and 48 h incubation was assessed using a Crystal Violet staining assay (Fig 5). Expression of *TL*Oβ*2* in strain Wü284 resulted in a significant decrease in biofilm formation at 24 h (Fig 5). Expression of *TLO*γ*11* and *TLO*α*12* resulted in a higher degree of biofilm formation at the 24 h timepoint (Fig 5). The greatest increase in biofilm formation relative to Wü284 was observed in the P_*ACT1*_-*TLO*γ*11* and P_*ACT1*_-*TLO* α*12* expressing strains, which exhibited increased biofilm at 24 h and 48 h (Fig 5).

### *TLO* expansion in wild type *C*. *dubliniensis* enhances virulence in the *Galleria mellonella* infection model

**Fig 6:**
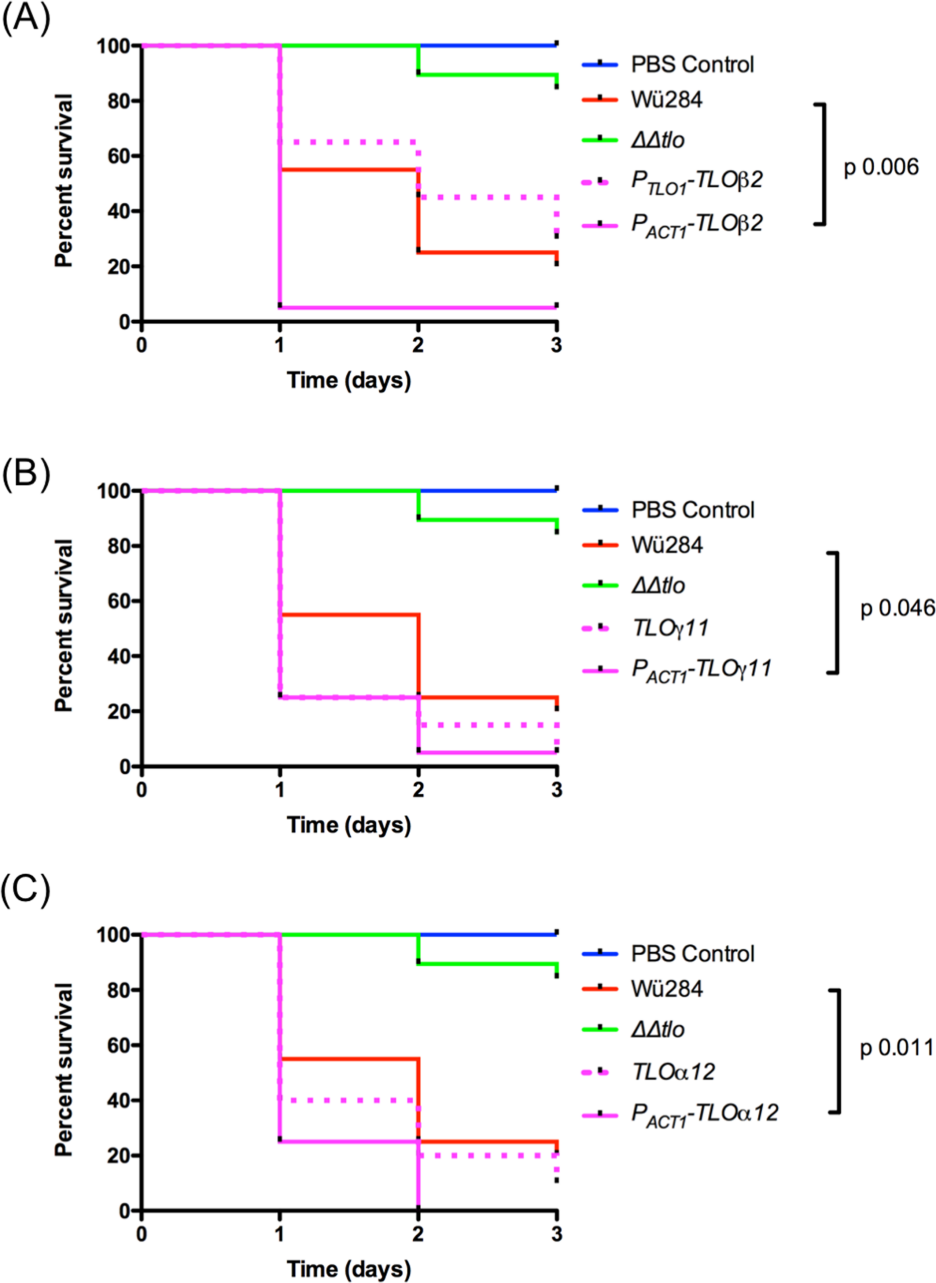
The effect of *C*. *albicans TLO* genes on the virulence of wild-type Wü284. Ten *G*. *mellonella* larvae were inoculated with 1 X 10^6^ cells of each indicated strain (performed blind) and viability was monitored over 3 days. Results presented represent three independent infection experiments. *P* values indicate results of a Log-Rank (Mantel-Cox) test against the wild type Wü284 survival curve.

Given the differential effects of specific *TLO* genes on various virulence attributes, such as morphology, stress tolerance and cell wall integrity, we decided to investigate if differences in virulence could be detected using an *in vivo* infection model. Virulence of WT *C*. *dubliniensis* Wü284 expressing *TL*Oβ*2*, *TL*Oγ*11* and *TL*Oα*12* from a native *TLO* promoter or the *ACT1* promoter was investigated using the insect larval *G*. *mellonella* model. Although genes expressed from native *TLO* promoters did not confer significant increases in virulence, expression of these *C*. *albicans TLO* genes in WT *C*. *dubliniensis* under the control of the *ACT1* promoter significantly enhanced virulence in this larval infection model (Fig 6). This effect was most significant in the case of the P_*ACT1*_- *TL*Oβ*2* expressing strain.

### Expression of *Candida albicans TLO* genes in the *C*. *dubliniensis Δtlo1/Δtlo2* (*ΔΔtlo*) double mutant

In order to further investigate the range of phenotypes regulated by *C*. *albicans* Tlo proteins, we also expressed representative members of the *C*. *albicans TLO* gene family in the *C*. *dubliniensis* ***Δ****tlo1/****Δ****tlo2* (***ΔΔ****tlo*) double mutant under the control of their native upstream regulatory elements. Several attempts were made to generate stable transformants expressing *TLO*β*2*, however, no viable transformants were recovered in these experiments. Quantitative Real Time PCR was used to determine the level of expression of the *C*. *albicans TLO*s in the *C*. *dubliniensis* ***ΔΔ****tlo* backgrounds (S1 Fig). *TLO 1*, *3*, *9* and *12*, all of which belong to the αclade, show similar expression levels of expression relative to *ACT1* (0.016 to 0.088, Fig 1B). Interestingly, the ɣclade genes *TLO*γ*7* (0.38 relative to *ACT1)* and *TLO*γ*11* (0.006 relative to *ACT1*) differed greatly in expression levels compared with one another (S1 Fig).

**Fig 7:**
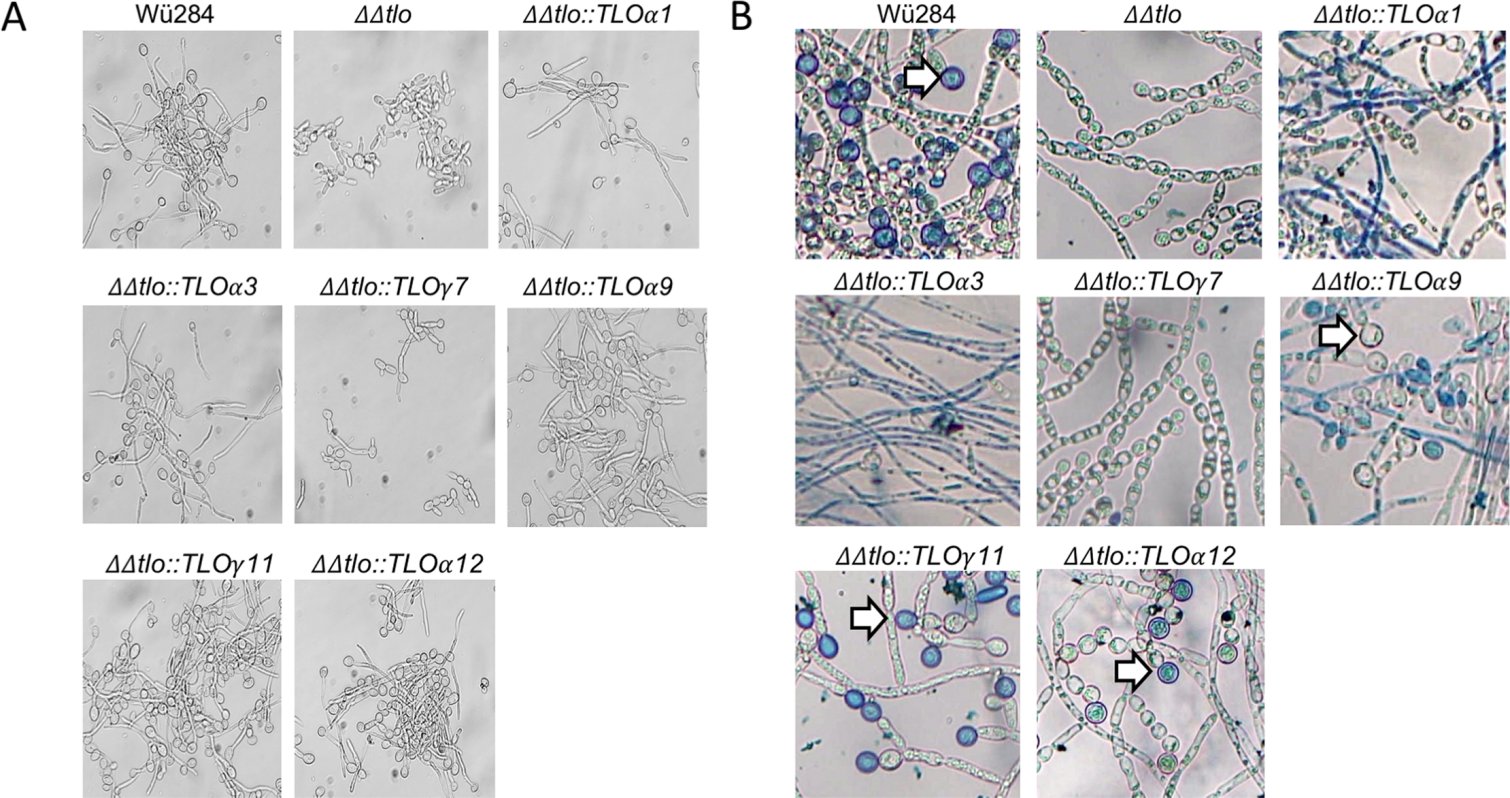
Morphology of *C*. *dubliniensis ΔΔtlo* expressing *CaTLO* genes. A. Photomicrographs of *C*. *dubliniensis* ***ΔΔ****tlo* and derivatives harboring the indicated *C*. *albicans TLO* genes following 4 h growth in water supplemented with 10% (v/v) foetal bovine serum at 37°C. B. Chlamydospore formation of *C*. *dubliniensis* ***ΔΔ****tlo* and derivatives harboring the indicated *C*. *albicans TLO* genes on cornmeal agar supplemented with tween. Chlamydospores are indicated by arrows. Identical results were observed in replicate experiments.

All *TLO* genes tested, with the exception of *TLO*γ*7*, restored filamentous growth in the *C*. *dubliniensis* ***ΔΔ****tlo* mutant, which is normally not capable of forming true hyphae (Fig 7A). *TLOα9*, γ*11* and *α12* restored the ability to produce chlamydospores in the deletion mutant, while *TLOα1*, *α3* and γ*7* were unable to do so (Fig 7B).

**Fig 8:**
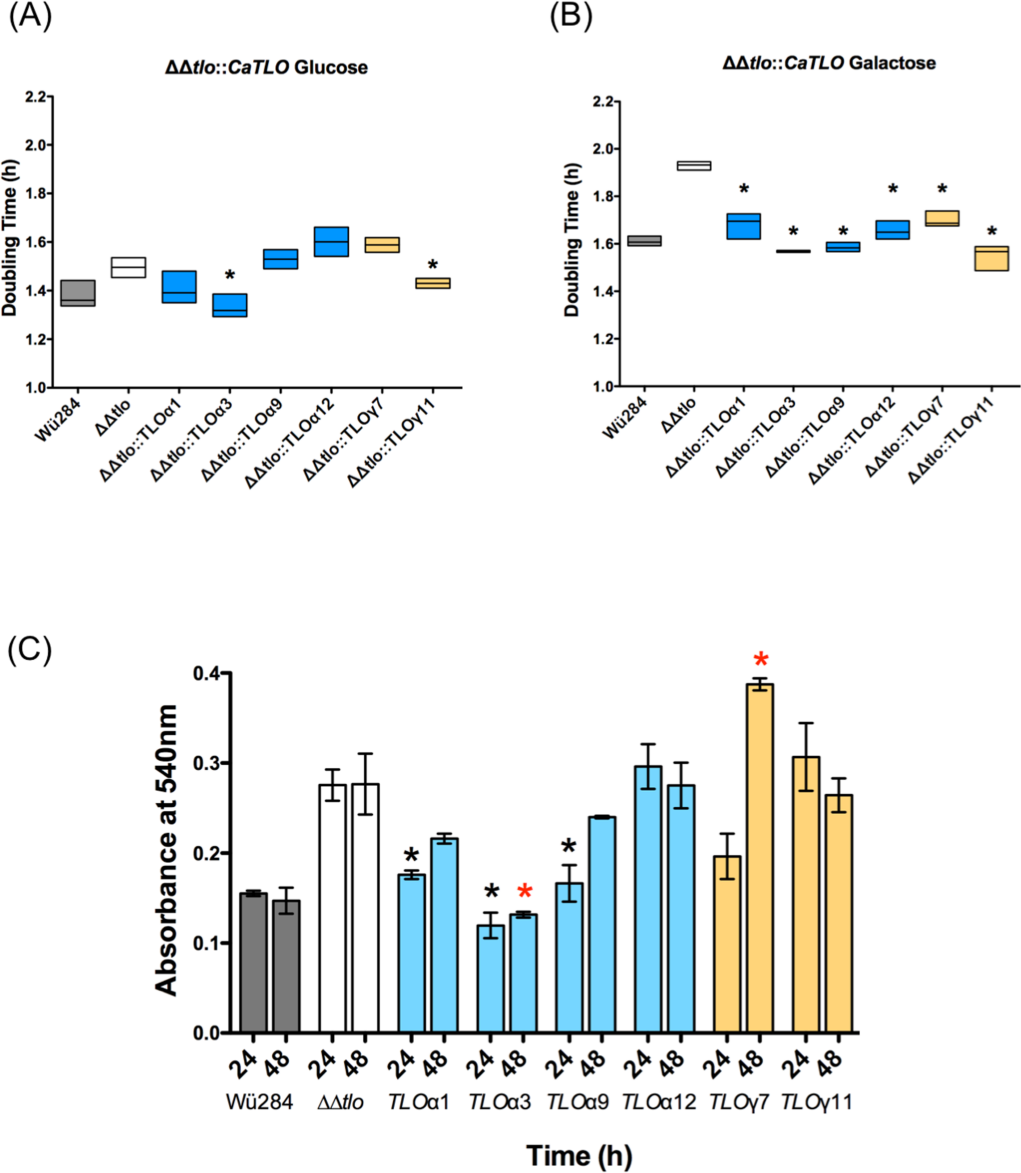
Growth of *C*. *dubliniensis ΔΔtlo* and derivatives harboring *C*. *albicans TLO* genes. A and B show doubling times of WT Wü284, the ***ΔΔ****tlo* double mutant and derivatives expressing indicated *C*. *albicans TLO* genes in YEP-Glucose (A) and -Galactose (B). Stars indicate strains exhibiting doubling times significantly different from ***ΔΔ****tlo* (p ≤ 0.05). Panel C shows biofilm formation on plastic surfaces. Each ***ΔΔ****tlo*::*TLO* strain was grown in the presence of YEPD in a 96-well plate for 48 h. Biomass was measured using a crystal violet assay in three replicate experiments. Asterisks indicate significant differences from ***ΔΔ****tlo* at 24 h (*) and 48 h (red *), respectively. Data are the result of three independent experiments.

All of the *TLO* genes complemented the defective growth of the ***ΔΔ****tlo* mutant in YEP-Galactose (Fig 8A and 8B). The ***ΔΔ****tlo C*. *dubliniensis* mutant has previously been shown to produce excess levels of biofilm on plastic surfaces relative to wild type [12]. Following 24 h growth under biofilm forming conditions, the ***ΔΔ****tlo*::*TLO*α*1*, *α3*, and *α9* strains exhibited reduced biofilm formation relative to the ***ΔΔ****tlo* double mutant and comparable to that observed with WT Wü284. The remaining genes tested either resulted in similar or greater (e.g. *TLO*γ*7*) levels of biofilm than the ***ΔΔ****tlo* double mutant (Fig 8C).

**Fig 9:**
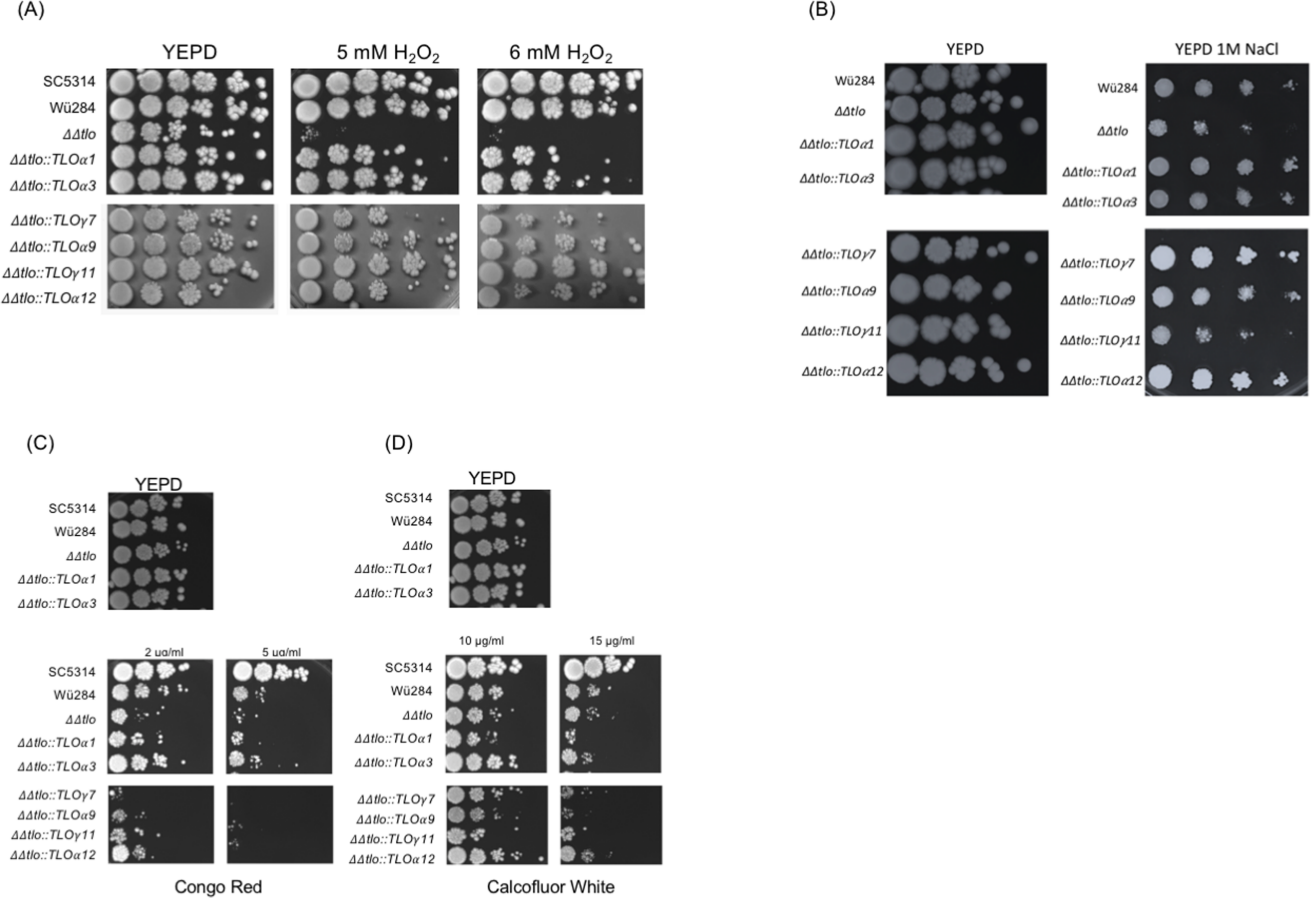
*C*. *albicans TLO* genes differentially affect tolerance of environmental stress conditions. Growth of each ***ΔΔ****tlo*::*CaTLO* strain in the presence (A) H_2_O_2_, (B) NaCl, (C) Congo Red and (D) Calcofluor White. Ten-fold serial dilutions (left to right) of 2 x 10^4^ cells were spotted onto YEPD agar and YEPD agar containing the indicated agents. Plates were incubated for 48 h at 37°C.

*TLO*γ*11* also conferred increased resistance to oxidative stress. At a concentration of 6 mM H_2_O_2_ ***ΔΔ****tlo*::*TLO*γ*11* had the greatest effect on enhancing tolerance of oxidative stress, with the remaining genes conferring tolerance, but to a lesser extent (Fig 9A). When incubated on solid YEPD supplemented with 1 M NaCl, all *CaTLO* genes tested, with the exception *CaTLO*γ*11*, resulted in increased growth compared to the ***ΔΔ****tlo* mutant strain (Fig 9B). In the presence of the cell wall perturbing compounds Congo Red (2 μg/ml) and Calcofluor White (10 μg/ml) *TLOα3* consistently restored growth of the ***ΔΔ****tlo* mutant to wild-type levels. ***ΔΔ****tlo*::*TL*O*α12* also exhibited enhanced levels of growth on Calcofluor White (10 μg/ml) compared to the ***ΔΔ****tlo* mutant (Fig 9C and 9D).

**Fig 10:**
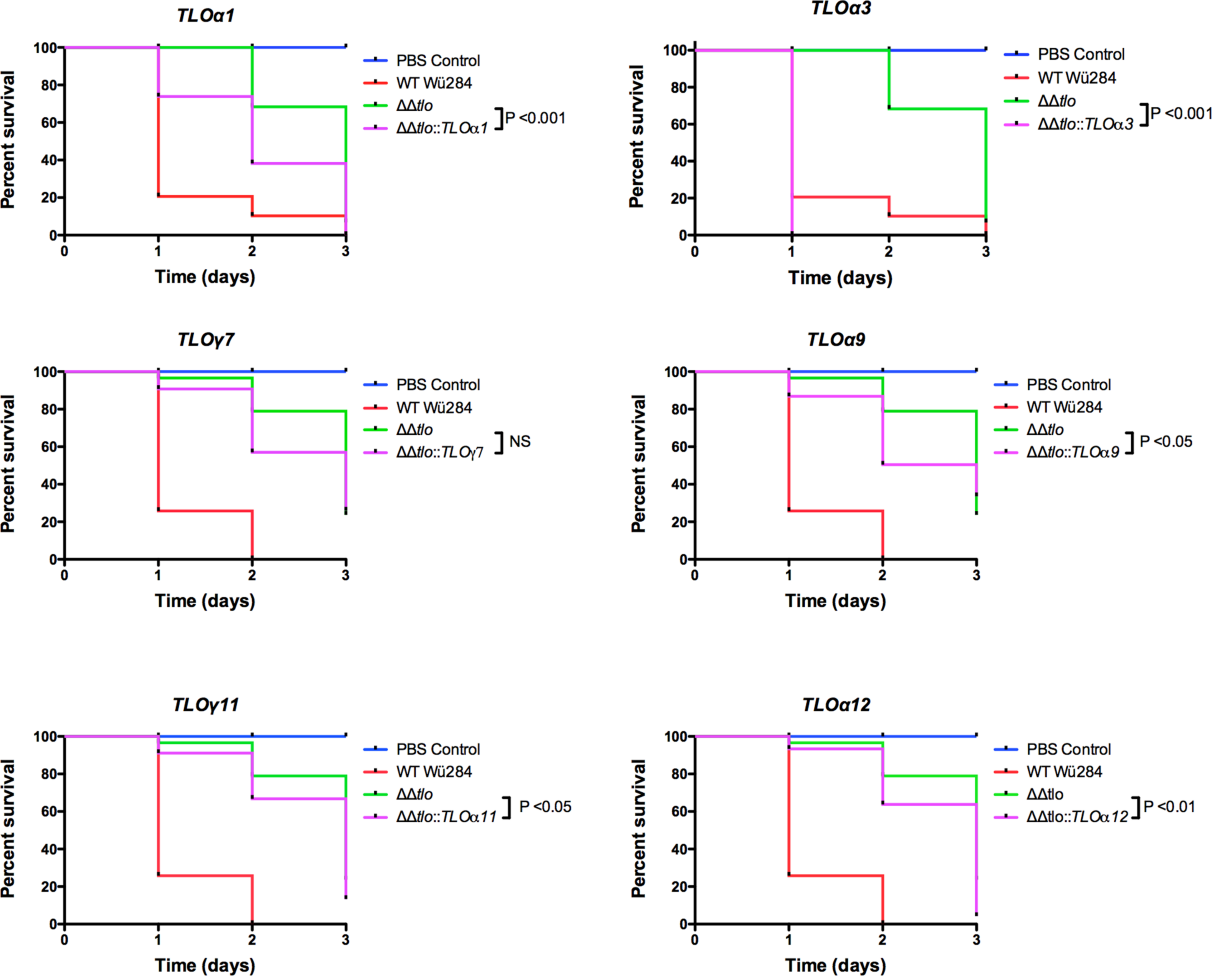
The effect of *C*. *albicans TLO* genes on the virulence of the *ΔΔtlo* mutant. Ten *G*. *mellonella larvae* were inoculated with 1 X 10^6^ cells of each indicated strain (performed blind) and viability was monitored over 3 days. Results presented represent three independent infection experiments. *P* values indicate results of a Log-Rank (Mantel-Cox) test against the ***ΔΔ****tlo* mutant survival curve.

Finally, examination of the virulence of the *CaTLO* expressing strains in the *Galleria mellonella* model showed that ***ΔΔ****tlo*::*TLO*α*3* enhanced virulence to the greatest extent, with survival rates significantly less than the parental ***ΔΔ****tlo* mutant and similar to the WT strain (Fig 10). The remaining *TLO* genes tested were shown to result in a restoration of virulence in the infection model with mortality rates greater than that of the ***ΔΔ****tlo* double mutant but less than the WT Wü284.

## Discussion

One of the largest gene families in *C*. *albicans* is the *TLO* family, which consists of up to 15 members, each encoding a protein orthologous to the Med2 subunit of the transcriptional regulator complex Mediator [28]. This expansion is unique to *C*. *albicans* and there is significant variation in the copy number of genes in this family between different strains [14]. There are only two *TLO* genes encoded in the genome of *C*. *dubliniensis*, the species most closely related species to *C*. *albicans*. Deletion of the two *TLO* genes in *C*. *dubliniensis* resulted in significant transcriptional and phenotypic defects, including an inability to produce hyphae and reduced tolerance of oxidative stress. Reintroduction of each of the *C*. *dubliniensis TLO* genes into the double mutant background indicated that the *CdTLO1* and *CdTLO2* genes differ in their ability to complement the mutant phenotypes, suggesting they may have distinct functions in gene control [12]. The purpose of the current study was to investigate if expansion of this two-membered family in *C*. *dubliniensis* affects phenotypes associated with virulence. Such a finding would lend support to our hypothesis that expansion of the *C*. *albicans TLO* gene family played a role in the evolution of the enhanced virulence of this species in comparison with other related species.

**Fig 11:**
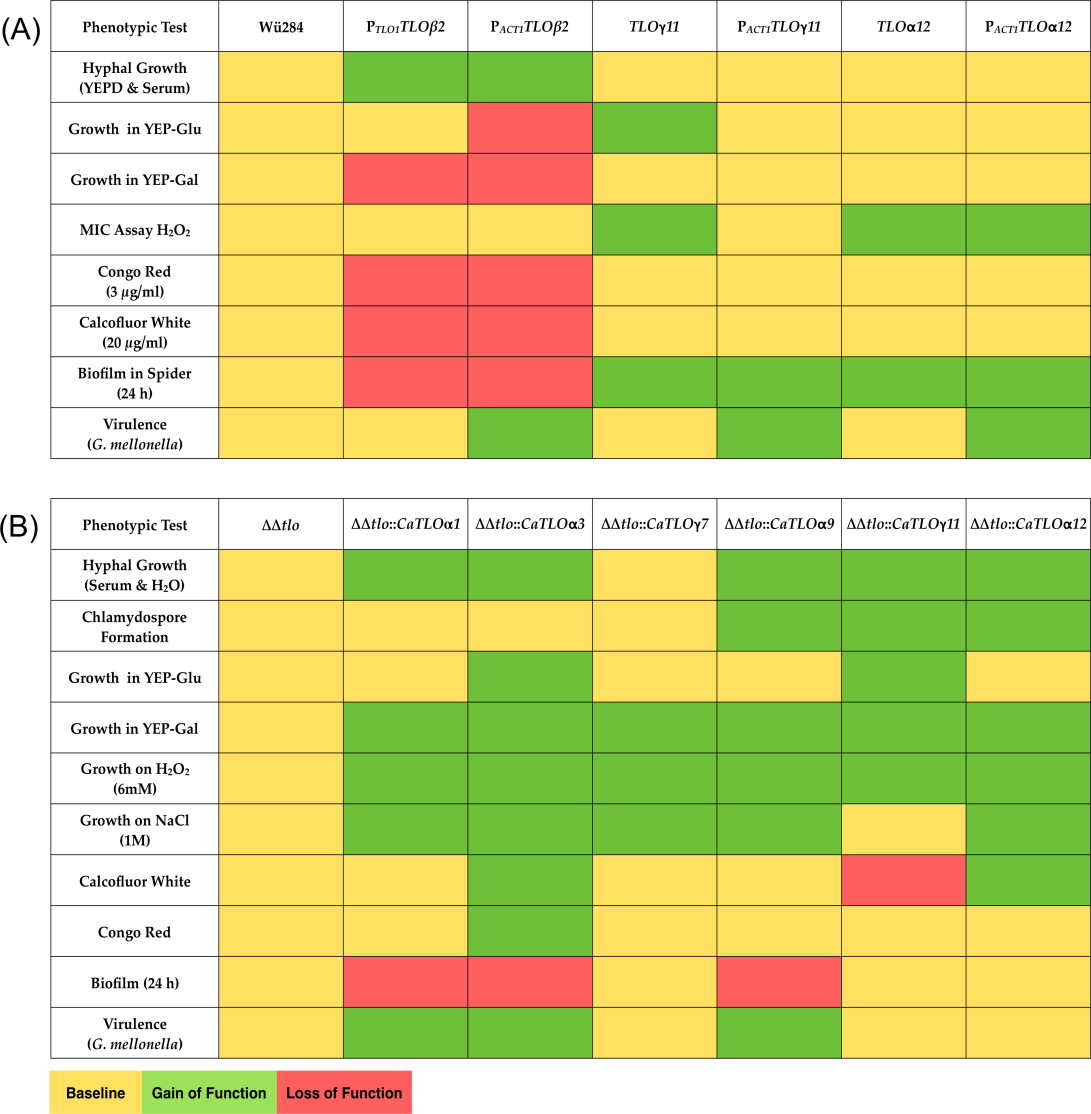
Heat maps summarizing phenotypic effects of *CaTLO* genes. The phenotypic effects of expressing each *CaTLO* gene in the *C*. *dubliniensis* wild-type (panel A) and in *C*. *dubliniensis* ΔΔ*tlo* (panel B) are colour coded; yellow indicates the same phenotype as the mutant, green indicates a gain of function and red indicates a loss of function.

We expanded the repertoire of Tlo proteins in *C*. *dubliniensis* Wü284 using representatives of the α, β, and γ *TLO* families, namely *TLOβ2*, *TLOγ11* and *TLOα12* and a summary of the effects of this expansion is shown in Fig 11A. Expression of *TLOβ2* had the most dramatic effect on morphology, resulting in the production of wrinkled colonies containing cells with hyphal morphologies. Interestingly, the extent of this phenotype was influenced by the expression level of *TLOβ2*, with the *ACT1*-promoter driven gene resulting in more highly-wrinkled colonies with a higher proportion of true hyphae. The more pronounced phenotype in the highly expressed construct indicates that expression level influences phenotype. This affect may be exerted by displacing endogenous CdTlo1 and CdTlo2 from the Mediator complex, therefore promoting Tloβ2 regulated functions. Alternatively, the higher expression levels may create a pool of Tlo in excess of Mediator. A similar phenotype was recently described following overexpression of CdTlo2 to create a Mediator excess population of Tlo in *C*. *dubliniensis* [20]. Unexpectedly, *TLOβ2* could not be expressed in the *C*. *dubliniensis* ΔΔ*tlo* double mutant, suggesting that a Mediator complex exclusively containing Tloβ2 is lethal to the cell.

Although wild-type Wü284 expressing *TLOγ11* and *TLO*α*12* were not affected in morphology, these strains exhibited a specific enhanced resistance to H_2_O_2,_ whereas the *TLOβ2* expressing strains which were filamentous were highly susceptible to oxidative and cell wall stress, suggesting that the morphology of the cells may contribute to their ability to tolerate stress. Expression of *TLOβ2*, *TLOγ11* and *TLOα12* in wild type Wü284 had varying effects on growth rates *in vitro*. *TLOβ2* generally increased doubling times in YEP-Glucose and -Galactose and this might be related to the hyper-filamentous, polarised growth pattern exhibited by this strain. In contrast, *TLOγ11* transformants exhibited a small (approximately 10 min) but significant reduction in doubling time in YEP-Glucose.

Infection of *G*. *mellonella* larvae with these strains showed that expression of the heterologous genes at low level using native *TLO* promoters had limited effects on larval survival, however expression under the control of the stronger *ACT1* promoter resulted in significantly reduced larval survival (similar to *C*. *albicans* SC5314). These data for the first time provide experimental evidence supporting enhanced fitness as a result of *TLO* copy number expansion in *Candida species*. It is interesting to note that the phenotypic effect was most significant when the genes were expressed under the control of the stronger *ACT1* promoter, indicating that a critical level of Tlo is required for this gain of function. This may partly explain why *C*. *albicans* has expanded the *TLO* family to such a significant extent.

In the second part of our study, to better understand the diversity of functions regulated by *TLOs*, we expressed a range of *C*. *albicans TLO* genes in a ΔΔ*tlo C*. *dubliniensis* background. In general, heterologous expression of the *C*. *albicans TLO* genes in the ΔΔ*tlo C*. *dubliniensis* background could restore all phenotypes examined, including some subtle and some major differences in the ability of individual paralogs to complement the phenotypes. A summary of these phenoptypes is shown in Fig 11B. It is clear from this heatmap that individual *CaTLO*s differ in their ability to affect specific phenotypes and in the magnitude of their restorative capability. For example, *TLOα1*, *α3* and *γ7* do not have the ability to restore chlamydospore production in the mutant, while *TLOγ11* and *α12* did not suppress biofilm formation (despite being active inducers of stress responses). The exception to this was *TLOγ7*, which despite having the highest expression level of all of the *C*. *albicans TLO*s (0.38 relative to *ACT1*) tested in the ΔΔ*tlo C*. *dubliniensis* background, had the least effect on restoring the phenotypes in the mutant. *TLOγ7* failed to restore the ability to form hyphae and chlamydospores or growth in media containing cell perturbing compounds. However, *TLOγ7* did restore growth in YEP-Gal and tolerance of sodium chloride and H_2_O_2_, indicating that the gene possesses some functionality. In order to investigate if there are differences in the effects of specific *CaTLO* genes on virulence we tested the virulence of strains in the *Galleria mellonella* larval infection model. *TLOα3* was found to restore virulence in the *C*. *dubliniensis* ΔΔ*tlo* mutant to a greater extent than the other *TLO* genes tested, suggesting a clear disparity in the activity of specific *TLO* genes.

These data complement the findings of Dunn *et al*. [21] who used a ‘Tet-ON’ missexpression system to probe individual *TLO* genes in *C*. *albicans* SC5314. The authors concluded that *TLO*s controlled multiple phenotypes and that single phenotypes were often regulated by multiple *TLO*s, including virulence in *G*. *mellonella*. However, it is difficult to directly compare the results of individual phenotypic tests in both studies due the different nature of the host strains (*C*. *albicans* and *C*. *dubliniensis*) and the phenotypic tests used.

In summary, the *C*. *albicans TLO* gene family is comprised of fifteen genes, mainly situated in the subtelomeric region of the chromosomes. These regions have been shown in other organisms to undergo rapid evolution and gene families in the subtelomeres have been demonstrated to expand rapidly and undergo functional divergence [29]. Our data support the hypothesis that there is functional diversity in the *C*. *albicans TLO* gene family and also indicate that the high copy number of *TLO* genes in C. *albicans* may have evolved to increase gene dosage, which in our larval infection model has a significant effect on virulence. Studies are now underway to confirm these hypotheses by attempting to deplete the *TLO* gene family in *C*. *albicans* using CRISPR-Cas9 mutagenesis.

## Supporting information

S1 Fig Expression of *CaTLO* genes in *C*. *dubliniensis* the ΔΔ*tlo* mutant. RT-PCR expression data of respresentative *C*. *albicans TLO* genes expressed in the *C*. *dubliniensis* ΔΔ*tlo* mutant strain. RT-PCR expression graphs represent three independent experiments.
(TIF)

S1 Table List of strains used in this study. (DOCX)

S2 Table Sequence of oligonucleotides used in this study. (DOCX)

S1 File DNA sequence of P_*TLO1*_*-TLOβ2* gene fusion. (TXT)
