# Supplementary figures and images for: Expansion of the TLO gene family enhances the virulence of Candida species
Source: PLoS One. 2018 Jul 20;13(7):e0200852. doi: 10.1371/journal.pone.0200852 (PMC6054389; doi:10.1371/journal.pone.0200852)

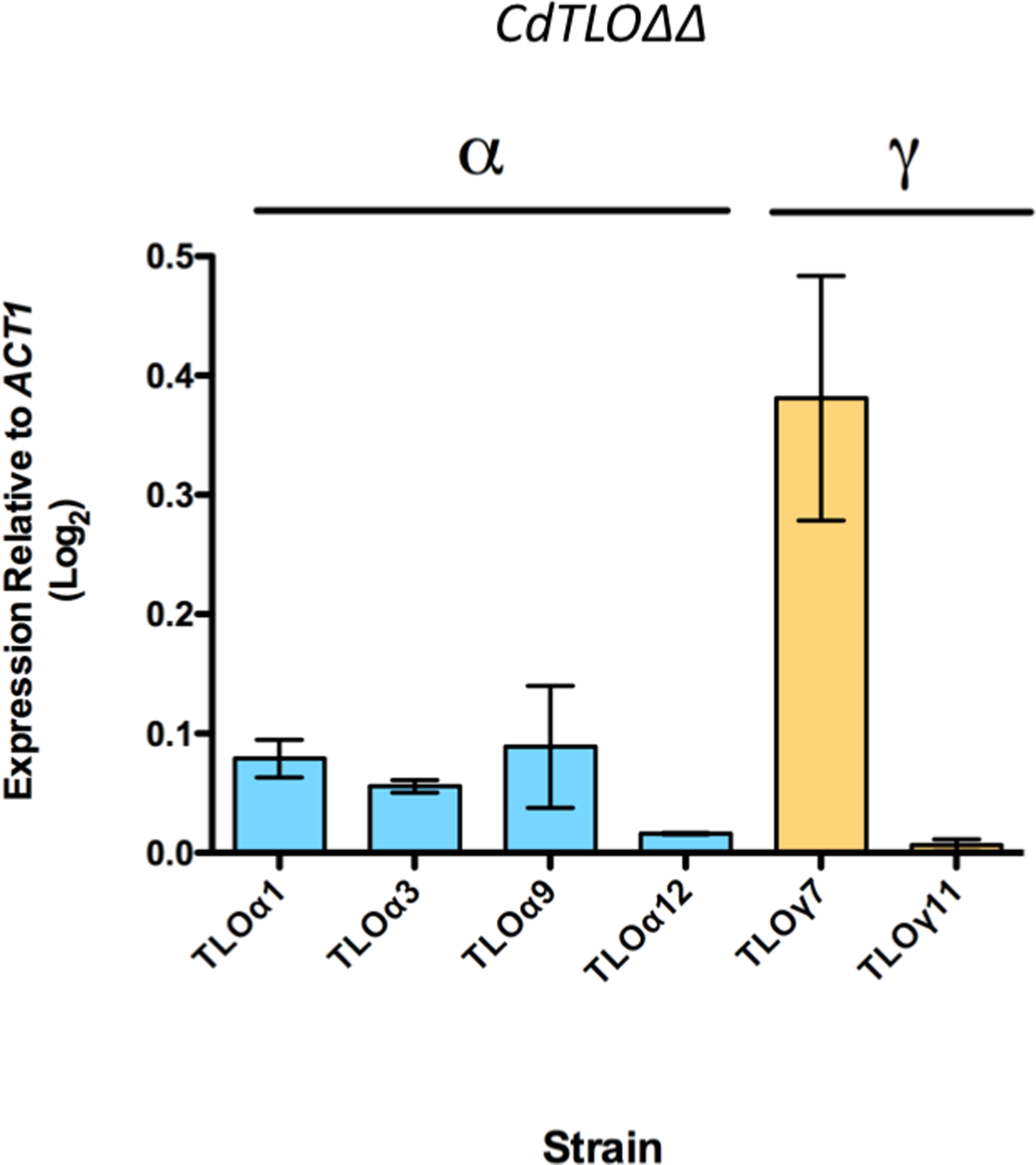

Supplement: S1 Fig — RT-PCR expression data of respresentative C. albicans TLO genes expressed in the C. dubliniensis ΔΔtlo mutant strain. RT-PCR expression graphs represent three independent experiments. (TIF) [file pone.0200852.s001.tif]
