# Supplementary material for: Expansion of the TLO gene family enhances the virulence of Candida species
Source: PLoS One. 2018 Jul 20;13(7):e0200852. doi: 10.1371/journal.pone.0200852 (PMC6054389; doi:10.1371/journal.pone.0200852)
Supplement: S1 Table — (DOCX) [file pone.0200852.s002.docx]

**S1 Table. List of strains used in this study**

| **Strain**  **Name** | **Parent Strain** | **Genotype** | **Reference** |
| --- | --- | --- | --- |
| **SC5314** | N/A | Wild-type *C. albicans* | (1) |
| **Wü284** | N/A | Wild-type *C. dubliniensis* | (2) |
| **∆∆*tlo*** | Wü284 | ∆*tlo1*::FRT/∆*tlo1*::FRT;∆tlo2::FTR* | (3) |
| **∆∆*tlo*::*CaTLOα1*** | ∆∆*tlo* | ∆*tlo1*::FRT/∆*tlo1*::FRT; ∆*tlo2*::FRT*; *CDR1*/*cdr1*::pCDRI-*CaTLO1* | This  Study |
| **∆∆*tlo*::*CaTLOα3*** | ∆∆*tlo* | ∆*tlo1*::FRT/∆*tlo1*::FRT; ∆*tlo2*::FRT*; *CDR1*/*cdr1*::pCDRI-*CaTLO3* | This  Study |
| **∆∆*tlo*::*CaTLOγ7*** | ∆∆*tlo* | ∆*tlo1*::FRT/∆*tlo1*::FRT; ∆*tlo2*::FRT*; *CDR1*/*cdr1*::pCDRI-*CaTLO7* | This  Study |
| **∆∆*tlo*::*CaTLOα9*** | ∆∆*tlo* | ∆*tlo1*::FRT/∆*tlo1*::FRT; ∆*tlo2*::FRT*; *CDR1*/*cdr1*::pCDRI-*CaTLO9* | This  Study |
| **∆∆*tlo*::*CaTLOγ11*** | ∆∆*tlo* | ∆*tlo1*::FRT/∆*tlo1*::FRT; ∆*tlo2*::FRT*; *CDR1*/*cdr1*::pCDRI-*CaTLO11* | This  Study |
| **∆∆*tlo*::*CaTLOα12*** | ∆∆*tlo* | ∆*tlo1*::FRT/∆*tlo1*::FRT; ∆*tlo2*::FRT*; *CDR1*/*cdr1*::pCDRI-*CaTLO12* | This  Study |
| **Wü::P*_ACT_TLOβ2*** | Wü284 | *CDR1*/*cdr1*::pGM161-P*_ACT_TLOβ2* | This  Study |
| **Wü::P*_TLO1_TLOβ2*** | Wü284 | *CDR1*/*cdr1*:: pCDRI-P*_TLO1_TLOβ2* | This  Study |
| **Wü::P*_ACT_TLOγ11*** | Wü284 | *CDR1*/*cdr1*::pGM161-P*_ACT_TLOγ11* | This  Study |
| **Wü::*TLOγ11*** | Wü284 | *CDR1*/*cdr1*::pCDR1-*CaTLOγ11* | This  Study |
| **Wü::P*_ACT_TLOα12*** | Wü284 | *CDR1*/*cdr1*:: pGM161-P*_ACT_TLOα12* | This  Study |
| **Wü::*TLOα12*** | Wü284 | *CDR1*/*cdr1*:: pCDRI-*CaTLOα12* | This  Study |

*Only one allele of *TLO2* was detected in Wü284

1. **Gillum AM**, **Tsay EY**, **Kirsch DR**. 1984. Isolation of the Candida albicans gene for orotidine-5'-phosphate decarboxylase by complementation of S. cerevisiae ura3 and E. coli pyrF mutations. Mol Gen Genet **198**:179–182.

2. **Morschhäuser J**, **Ruhnke M**, **Michel S**, **Hacker J**. 1999. Identification of CARE-2-negative Candida albicans isolates as Candida dubliniensis. Mycoses **42**:29–32.

3. **Haran J**, **Boyle H**, **Hokamp K**, **Yeomans T**, **Liu Z**, **Church M**, **Fleming AB**, **Anderson MZ**, **Berman J**, **Myers LC**, **Sullivan DJ**, **Moran GP**. 2014. Telomeric ORFs (TLOs) in Candida spp. Encode Mediator Subunits That Regulate Distinct Virulence Traits. PLoS Genet **10**:e1004658.
