# Supplementary material for: Expansion of the TLO gene family enhances the virulence of Candida species
Source: PLoS One. 2018 Jul 20;13(7):e0200852. doi: 10.1371/journal.pone.0200852 (PMC6054389; doi:10.1371/journal.pone.0200852)
Supplement: S2 Table — (DOCX) [file pone.0200852.s003.docx]

**S2 Table. Sequence of oligonucleotides used in this study**

| Oligonucleotide Name | Oligonucleotide Sequence |
| --- | --- |
| *CaTLO1*_xhoIF | CCGCTCGAGTGATCATACATATCTTAACTTAG |
| *CaTLO1*_hindIIIR | GGGAAGCTTCTGTATATGTCTTGTAGAAC |
| *CaTLO3*_xhoIF | CCGCTCGAGTATAGCTAGATACGTGTA |
| *CaTLO3*_hindIIIR | GGGAAGCTTCGTATTCCTGTACCA |
| CaTLO4_hindIIIR | GGGAAGCTTAGAAAGCAGAGACCGC |
| CaTLO7_xhoIF | CCGCTCGAGGAATTAGAACACCAATC |
| CaTLO7_hindIIIR | GGGAAGCTTCTCCTCTGCCTTCTT |
| CaTLO8_xhoIF | CCGCTCGAGGCGACTTCTTAGT |
| CaTLO8_hindIIIR | GGAAGCTTGGTGTAGATGTCC |
| CaTLO9_xhoIF | CCGCTCGAGTTCGTAGTTGAAGAT |
| CaTLO9_hindIIIR | GGGAAGCTTGAACTTGTTGAACAG |
| CaTLO11_xhoIF | CCGCTCGAGTGGTGTCTTATAGAGAT |
| CaTLO11_hindIIIR | GGGAGCTTAAGAAGGCAGAGACCG |
| CaTLO12_xhoIF | CCGCTCGAGAACGACTATAAATTGAG |
| CaTLO12_hindIIIR | GGGAAGCTTATATATCAAGGTGCTATTTCC |
| TLO1_qRT F1 | ATATTAACATATCAAGGAG |
| TLO1_qRT R1 | CAATTATCTCAAATATGTATCC |
| TLO3_qRT F1 | TCCCTATATATCAAGGTGTTA |
| TLO3_qRT R1 | CGATTATCTCAAATATGTATCCT |
| TLO7_qRT F1 | TAACTAGCCCCAACAACGAAC |
| TLO7_qRT R1 | TTCGACACTGTTTGGTCTAGAA |
| TLO9_qRT F1 | CCTCCAAACAAGACTACATAAC |
| TLO9_qRT R1 | TCTTGTAGGACAGAGCCCC |
| TLO11_qRT F1 | TCATCAGGATACATATTTGAG |
| TLO11_qRT R1 | ACTTCTTGGCTTCCTCTGCT |
| TLO12_qRT F1 | ACTAGCCCCAACAACGAACT |
| TLO12_qRT R1 | ACGTTTCTTTCTTGCCGCTT |
| ACT F1 | AGCTCCAGAAGCTTTGTTCAGACCAG |
| ACT R1 | TGCATACGTTCAGCAATACCTGGG |
